# Efficacy of Commercial Sanitizers Used in Food Processing Facilities for Inactivation of *Listeria monocytogenes*, *E. Coli* O157:H7, and *Salmonella* Biofilms

**DOI:** 10.3390/foods8120639

**Published:** 2019-12-04

**Authors:** Manish Aryal, Peter M. Muriana

**Affiliations:** 1 Robert M. Kerr Food & Agricultural Products Center, Oklahoma State University, Stillwater, OK 74078-6055, USA; aryalm3@gmail.com; 2 Department of Animal and Food Sciences, Oklahoma State University, Stillwater, OK 74078-6055, USA

**Keywords:** biofilm, sanitizer, *L. monocytogenes*, *Salmonella*, *E. coli* O157:H7, microplate assay

## Abstract

Bacteria entrapped in biofilms are a source of recurring problems in food processing environments. We recently developed a robust, 7-day biofilm microplate protocol for creating biofilms with strongly adherent strains of *Listeria monocytogenes, Escherichia coli* O157:H7, and *Salmonella* serovars that could be used to examine the effectiveness of various commercial sanitizers. *Listeria monocytogenes* 99-38, *E.coli* O157:H7 F4546, and *Salmonella* Montevideo FSIS 051 were determined from prior studies to be good biofilm formers and could be recovered and enumerated from biofilms following treatment with trypsin. Extended biofilms were generated by cycles of growth and washing daily, for 7 days, to remove planktonic cells. We examined five different sanitizers (three used at two different concentrations) for efficacy against the three pathogenic biofilms. Quaternary ammonium chloride (QAC) and chlorine-based sanitizers were the least effective, showing partial inhibition of the various biofilms within 2 h (1–2 log reduction). The best performing sanitizer across all three pathogens was a combination of modified QAC, hydrogen peroxide, and diacetin which resulted in ~6–7 log reduction, reaching levels below our limit of detection (LOD) within 1–2.5 min. All treatments were performed in triplicate replication and analyzed by one way repeated measures analysis of variance (RM-ANOVA) to determine significant differences (*p* < 0.05) in the response to sanitizer treatment over time. Analysis of 7-day biofilms by scanning electron microscopy (SEM) suggests the involvement of extracellular polysaccharides with *Salmonella* and *E. coli,* which may make their biofilms more impervious to sanitizers than *L. monocytogenes.*

## 1. Introduction

Sanitary practices in food manufacturing facilities target the elimination of pathogens and reduction of contaminating microbiota that gain access to the processing environment, often from the raw material and food ingredients, but also from workers, drains, and ventilation ducts [1,2,3,4]. Manufacturing shifts may process raw food ingredients for up to 8 h continuously. During this time, microorganisms may find harborage on food processing equipment and establish biofilms that may become the foci of contamination for manufactured foods or be displaced to other environmental locations. *Listeria monocytogenes, Bacillus cereus, E. coli* O157:H7*, Salmonella* spp*., Pseudomonas* spp.*,* and *Staphylococcus aureus* have been documented to form biofilms on food and food contact surfaces [3,5,6]. Apart from a bacteria’s ability to initiate attachment, intrinsic factors related to the chemistry of foods and extrinsic factors, such as the food contact surface itself, can influence the level of attachment and biofilm formation [7,8,9]. Bacteria may be influenced by different ‘scale effects’ of surface topography (i.e., nano- vs. micro-scale) that may affect attachment [10]. Early attachment may not be a completely random process, but rather may involve preferential attachment to sites that improve the chance of sustaining the development of biofilm [11]. These may involve various adhering chemistries of the external bacterial architecture, such as hydrophilic/hydrophobic attractions from charged amino acids of flagella and pilli, or of carbohydrates and lipids [12,13,14]. However they may occur, biofilms in food manufacturing facilities can lead to contaminated foods, resulting in outbreaks and illnesses among consumers [15].

Sinde and Carballo [16] found that the degree of attachment and efficacy of sanitizers on surface-biofilms varied considerably between surface types that may consist of various materials, including stainless steel, glass, polyurethane, teflon, rubber, or wood [17,18]. In contrast, adherence characteristics could be strain-dependent, as Gamble and Muriana [19] found strongly adherent strains of *L. monocytogenes* that were equally capable of forming biofilms on stainless steel, rubber, plastic, and glass, while less adherent strains were less capable of adhering to those surfaces. The attachment of pathogenic bacteria on these surfaces serves as a reservoir of microbial contamination and poses a high risk in the production line [20]. This has raised strong concerns for food safety and it is indispensable to develop proper cleaning and disinfection procedures for biofilm prevention and control [21].

In food industries, the disinfection of surfaces or equipment is mostly done by use of sanitizers [22,23]. There are different types of sanitizers, which can be grouped broadly as oxidizing agents, surface active compounds, and iodophores [24]. Widely used sanitizers, including halogen-based compounds, peracetic acid (PAA), ozone, and hydrogen peroxide, fall within the group of oxidizing agents [23]. Surface active compounds, such as acid anionic compounds and quaternary ammonium compounds (QACs), are also used abundantly in food industries [24]. However, the thick biofilm matrix, comprised of fat, carbohydrates, nucleic acids and protein-based materials, limits the effectiveness of many sanitizers. Moreover, pH, temperature, contact time, water hardness, and concentration are also important factors influencing the effectiveness of disinfectants [25,26]. *Listeria monocytogenes* was shown to increase its resistance to QACs, chlorine and hydrogen peroxide when biofilm maturation time was increased [27]. Similarly, resistance of *E. coli* O26 clinical and cattle isolates to QACs and PAA-based sanitizers was observed when exposed for up to 2 min [28]. Some cells might have natural resistance and some might acquire resistance to the sanitizers through genetic exchanges or mutations [29]. Even more alarming are reports that suggest a correlation of biocide use with the development of antibiotic resistance [30,31]. Reviews by Kampf have shown that both Gram-negative and Gram-positive bacteria are equally capable of enhancing antibiotic resistance when subjected to biocidal use [32,33]. This is the reason the US Food and Drug Administration banned the use of triclosan and other active agents from antimicrobial soaps for use at home by the general population. These capabilities, possessed by some microbes, allow them to grow and persist despite the application of sanitizers. Thus, increased resistance to biocides such as sanitizers is a concern in food industries, hence the development of new control strategies is highly advocated [34].

Post-harvest processing by various industries (meat, dairy, vegetable) are known to have specific microbiota-associated biofilms. The dairy industry has bacteria associated with drains, where nutritious spilled milk or whey results in a good growth environment in drains, and problems have long been associated with tubing systems for pasteurization that have dead-end zones or difficult to clean by clean-in-place systems [35]. The vegetable and fruit juice industries often have acetic and citric acid acidified products, which could lead to acid-tolerant organisms contributing to biofilm formation [36]. Perhaps no food processing industry is as large and diverse as the meat industry, involving live animal operations, slaughter and beef carcass processing, to fabricated beef cuts, ground beef, and further processing as ready-to-eat (RTE) meats, all capable of being besieged by microbial biofilms [37].

We recently optimized a microplate method to facilitate the generation of extended biofilms produced by strongly adherent strains of *L. monocytogenes*, *Salmonella*, and *E. coli* O157:H7 and the subsequent recovery of remaining viable cells using enzymatic detachment [38]. The current work describes our evaluation of five commercial sanitizers comprising the aforementioned types of sanitizers against the biofilms of these three pathogens.

## 2. Materials and Methods

### 2.1. Bacterial Strains and Growth Conditions

*E. coli* O157:H7 F4546, *L. monocytogenes* 99-38, and *Salmonella* Montevideo FSIS 051 were previously screened by a microplate fluorescence adherence assay differentiating them from other strains by their high-level adherence to form biofilms [19,38,39]. Active cultures were grown in Brain Heart Infusion broth (BHI, Difco, BD Laboratories, Franklin Lakes, NJ, USA) in 9 mL tubes at 30 °C. Cultures were harvested by centrifugation (6000 × *g*, 5 °C) of 9 mL of fresh, overnight cultures and cell pellets were resuspended in 2–3 mL of fresh sterile BHI broth containing 10% glycerol. Cell suspensions were placed into glass vials and stored in an ultra-low freezer (−80 °C). Frozen stocks were revived by transferring 100 µL of the thawed cell suspension into 9 mL of BHI broth, incubating overnight at 30 °C, and sub-cultured twice before use. Microbial enumeration for all assays was carried out on Tryptic Soy Agar (TSA, Bacto, BD), and plated in duplicate.

### 2.2. Growth of Enhanced Biofilms in Microplates

Overnight cultures (~9 log CFU/mL) of *E. coli* O157:H7 F4546, *L. monocytogenes* 99-38, and *S.* Montevideo FSIS 051 were diluted to ~4 log CFU/mL in BHI broth, and 200 µL was added to sterile Falcon 96-well clear, non-treated, flat-bottomed polystyrene microplates (Cat# 351172, Corning, NY, USA) to initiate biofilms. Inoculated plates were incubated at 30 °C, washed daily with the microplate washer, and fresh sterile media was added; this cycle of growth, wash, and media renewal was continued for 7 consecutive days in order to generate a robust ‘extended’ biofilm [38]. Microplate biofilms were washed in a plate washer to wash away planktonic cells and loosely-adhered cells, in addition to re-suspending settled planktonic cells before further use (described below).

### 2.3. Washing Biofilms Generated in Microplates

The microplates used for growing biofilms, and subsequently for sanitizer lethality assay and the detachment of remaining viable cells, were subjected to a wash treatment in a Biotek Elx405 Magna plate washer (Ipswich, Suffolk, UK). This plate washer was connected to separate wash (10% disinfectant bleach solution, sterile de-ionized water, or sterile 0.05 M Tris buffer pH 7.4, depending on the need) and waste containers. The plate washer has 96 pairs of needles (a longer one for aspiration and a shorter one for dispensing) to draw liquids into, and out of, each of the wells, and a shake parameter to shake the plate to re-suspend settled cells, or release loosely adhered cells, before washing. Before washing the 96-well microplates to which the bacteria were adhered, maintenance cycles were performed to sanitize the plate washer needles and tubing by washing with 10% disinfectant bleach (two times), followed by de-ionized water (three times), and Tris buffer (two times). After the rinses, microplates with adhered cells were washed with 0.05 M Tris buffer (pH 7.4) three times, each time using the shake option in the Elx405 plate washer.

### 2.4. Enzymatic Detachment of Adhered Cells from Microplates for Enumeration

A trypsin enzyme solution (Cat: T4549; 1486 U/mL; Sigma-Aldrich, St. Louis, MO, USA) of 500 U/mL from porcine pancreas was used to release adhered *L. monocytogenes*, *E. coli*, and *Salmonella,* in order to obtain a plate count enumeration of biofilm-adhered bacteria, either before (controls) or after (experimental) sanitizer treatment. After the final wash with 0.05 M Tris buffer (pH 7.4), 200 µL of trypsin enzyme solution was transferred into the experimental wells. After the addition of enzyme, the microplate was incubated for 1 h at 37 °C. Finally, to get detached cell counts, the solutions from the wells were surface plated on TSA plates and incubated at 30 °C for 24–36 h. The limit of detection (LOD) of plate counts from microplate wells was observed at 2 log CFU/mL (1:10 dilution of trypsinized cells from microplate wells, followed by surface plating of 0.1 mL).

### 2.5. Sanitizers Used in the Microplate Biofilm Assay

Different common and new generation sanitizers (Bi-Quat, 10-Chlor, Sterilex, KC-610, and Decon7) were used in this study to analyze their effects on biofilms (Table 1).

Bi-Quat (Birko, Henderson, CO, USA) was used at a concentration of 200 ppm (i.e., 0.08 gal per 40 gal of water, or 2 mL per 1 l of water) and 1000 ppm. The effects of Bi-Quat on pathogenic biofilms were observed over the time periods of 0, 15, 30, 60, and 120 min.

Chlorine-based 10-Chlor (Birko; 10% sodium hypochlorite) was used in two different concentrations of 200 ppm (2.5 oz. per 10 gal of water) and 1000 ppm (12.5 oz. per 10 gal of water). The biofilms were separately incubated with 200 ppm and 1000 ppm of 10-Chlor for five different time periods: 0, 5, 15, 30, and 60 min.

Sterilex solution (Sterilex Corporation, Cockeysville, MD, USA) is a two part liquid concentrate mixed together at the time of use. The two different parts are: Part 1 (Ultra Disinfectant Cleaner Solution 1) and Part 2 (Ultra Activator Solution). We used two different concentrations of working Sterilex sanitizer solution, 5% and 10%. The biofilm treatment time periods were 0, 1, 2.5, 5, 10, and 20 min for the 10% solution, and 0, 2.5, 5, 10, and 20 min for 10% solution.

Decon7 solution (Decon^™^ Seven Systems, Scottsdale, AZ, USA) came in three parts: Part 1—a surfactant (quaternary ammonium compound); Part 2—an oxidizer (hydrogen peroxide); and Part 3—an accelerator (diacetin). These three parts were mixed in the ratio 2:2:1 to form the stock solution. Working solutions were made at 5% and 10% concentrations of the stock solution to assess efficacy against biofilms. Similar to Sterilex solutions, the 5% Decon7 solution had treatment time periods of 0, 2.5, 5, 10, and 20 min, while the 10% Decon7 solution was used with treatment times of 0, 1, 2.5, 5, 10, and 20 min.

KC-610 (Packers Chemical, Kieler, WI, USA) is a peroxyacetic acid (PAA)-based antimicrobial solution, which was used as per the manufacturer’s instructions at a concentration of 6.1 oz. per 6.0 gal of water. The active ingredients of the solution were 5.6% peroxyacetic acid and 26.5% H_2_O_2._ The treatment time periods for this chemical were assigned at 0, 5, 15, 30, and 60 min.

### 2.6. Microplate Biofilm Sanitizer Assay

Biofilm lethality assays using various sanitizers were carried out in 96-well microplates. *Listeria monocytogenes* 99-38, *E. coli* O157:H7 F4546, and *S.* Montevideo FSIS 051 were used to form 7 day old mature biofilms (microplates were washed daily, sterile media replaced so only the adhered cells contribute to further growth). The 7-day biofilms were washed 3× with Tris buffer (0.05 M, pH 7.4) in the plate washer (with shaking) and 250 µL of different concentrations of various sanitizers were added (or Tris buffer for controls). After the sanitizer (or buffer) incubation periods, the microplates were again washed with Tris buffer, aspirated, and then 250 µL Dey-Engley (DE) neutralizing buffer (Hardy Diagnostics, Santa Maria, CA, USA) was added to the wells and left for 5 min to neutralize the effects of sanitizers. The microplates were then washed with Tris buffer (0.05 M. pH 7.4) in the plate washer, and 250 µL of trypsin (500 U/mL) was added into the wells and incubated for an hour at 37 °C. The solution from the trypsin-treated biofilm-containing wells was harvested and plated on TSA plates. The plates were then incubated for 24–36 h at 30 °C and enumerated the next day (24–30 h).

### 2.7. Scanning Electron Microscopy (SEM) of Biofilms

Biofilms of the three pathogens used in this study were examined by scanning electron microscopy (SEM) by inoculating ~4 log CFU/mL in BHI broth (250 µL) into wells of Millicell EZ Slide 8-well glass slides (Millipore Sigma, Sheboygan Falls, WI, USA), sealed with parafilm to avoid evaporation, and incubated at 30 °C; the media (BHI) and planktonic cells in the wells were manually removed, washed, and replenished each day, as described earlier for microplates, in order to achieve 7 day extended biofilms in the wells. A standard protocol provided by Oklahoma State University’s Electron Microscopy lab was used to fix, dry, and coat the samples before imaging. Cells were fixed for 2 h in 2.0% glutaraldehyde in 0.1 M cacodylate buffer (21.4 g sodium cacodylate brought to 500 mL with deionized H_2_O). The slides were then rinsed 3× in buffered wash (60 mL of 0.2 M cacodylate buffer, 140 mL of dH_2_O, and 12.3 g of sucrose; 15 min/rinse). Adherent cells were again fixed for 1 h in 1% aqueous osmium tetroxide (OsO_4_) at room temperature and then rinsed 3× in buffered wash solution (15 min/rinse). This was followed by dehydration in ethanol of different concentrations: 50%, 70%, 90%, 95%, and 100% (3×, 15 min/step), and then the slide(s) were subjected to critical point drying (CPD) or washed 2× for 5 min with HMDS (Hexamethyldisilazane). Silver paint or double-sticky tape was used to mount on stubs, which were then coated with gold–palladium (Au–Pd) and either visualized or stored in a dust-free dry area (desiccator) to view later. Visualization of the biofilms was done using an FEI Quanta 600 FEG scanning electron microscope (SEM) at the Oklahoma State University Electron Microscopy Core Facility.

### 2.8. Statistical Analysis

Each trial was performed in triplicate replication and all replications were performed as autonomous and separate experiments using separately inoculated cultures and prepared plating media. All data were presented as the mean of triplicate replications and standard deviation of the mean is represented by error bars. Statistical analysis of timed-series plots was done by repeated measures one way analysis of variance (RM-ANOVA) using the Holm–Sidak test for pairwise multiple comparisons to determine significant differences of sanitizer treatment on biofilms over time. Data treatments with different letters are significantly different (*p* < 0.05); treatments with the same letter are not significantly different (*p* > 0.05).

## 3. Results

### 3.1. Sanitizer Biofilm Microplate Assays vs. L. monocytogenes, Salmonella Montevideo, and E. coli O157:H7

#### 3.1.1. Hypochlorite-Based Sanitizer

**Figure 1:**
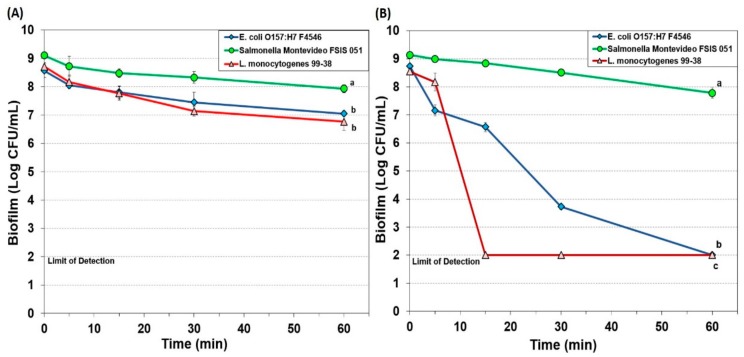
Biofilm microplate assay of *E. coli* F4546*, S.* Montevideo FSIS 051*, and L. monocytogenes* 99-38 against 7-day biofilms challenged with 10-Chlor sanitizer for 0–60 min at either 200 ppm (**A**) or 1000 ppm (**B**). Data points represent the means of triplicate replications and error bars represent the standard deviations from the mean (some error bars may be hidden by the large symbols). Treatments with different letters are significantly different (repeated measures (RM)-ANOVA, *p* < 0.05); treatments with the same letters are not significantly different (RM-ANOVA, *p* > 0.05).

A hypochlorite-based sanitizer (10-Chlor) was used against 7-day biofilms of *E. coli* F4546, *S.* Montevideo FSIS 051, and *L. monocytogenes* 99-38 at two different concentrations (200 and 1000 ppm) with plate counts representing trypsin-recovered cells after 0, 5, 15, 30, and 60 min treatment time (Figure 1). The use of 200 ppm 10-Chlor resulted in a minimum reduction of all three pathogens even when used for as long as 60 sec of immersion (Figure 1A). However, when levels were increased to 1000 ppm, *L. monocytogenes* dropped to undetectable levels between 5 and 15 min, *E. coli* F4546 slowly dropped to undetectable levels by 60 min, while *Salmonella* was not affected much more than it was at 200 ppm (Figure 1B).

#### 3.1.2. Simple Quaternary Ammonium Chloride-Based Sanitizer

**Figure 2:**
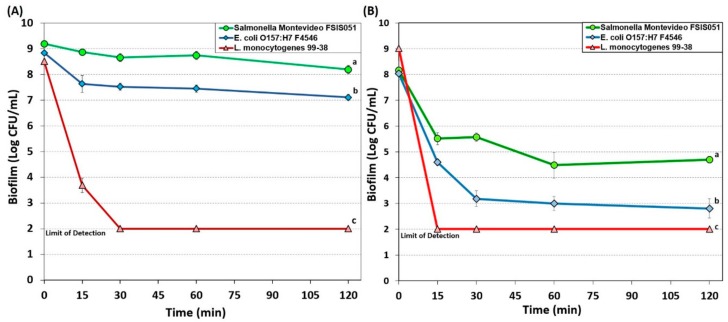
Biofilm microplate lethality assay of *E. coli* F4546*, S.* Montevideo FSIS 051*, and L. monocytogenes* 99-38 on 7-day extended biofilms challenged with Bi-Quat sanitizer for 0–120 min at 200 ppm (**A**) and 1000 ppm (**B**). Data points represent the means of triplicate replications and error bars represent the standard deviations from the mean (some error bars may be hidden by the large symbols). Treatments with different letters are significantly different (RM-ANOVA, *p* < 0.05); treatments with the same letters are not significantly different (RM-ANOVA, *p* > 0.05).

A simple QAC sanitizer (Bi-Quat) was used at 200 (Figure 2A) and 1000 ppm (Figure 2B) on biofilms of the three pathogens for 0, 15, 30, 60, and 120-min. Biofilms of *L. monocytogenes* 99-38 was most sensitive to Bi-Quat at both concentrations, and readily demonstrated nearly a 5 log decrease with 200 ppm within 15 min (Figure 2A); at 1000 ppm, a >7 log decrease was observed with the same treatment time (Figure 2B). However, similar to the situation with 10-Chlor, *E. coli* O157:H7F4546 and *S.* Montevideo FSIS 150 were more resistant to Bi-Quat, barely showing a 1 and 2 log reduction, respectively, with 200 ppm after 2 h treatment time (Figure 2A). Although 1000 ppm was effective in providing greater reductions of *Salmonella* and *E. coli* O157:H7, it did not completely inactivate them in biofilms (Figure 2B).

#### 3.1.3. Peroxyacetic Acid-Based Sanitizer

**Figure 3:**
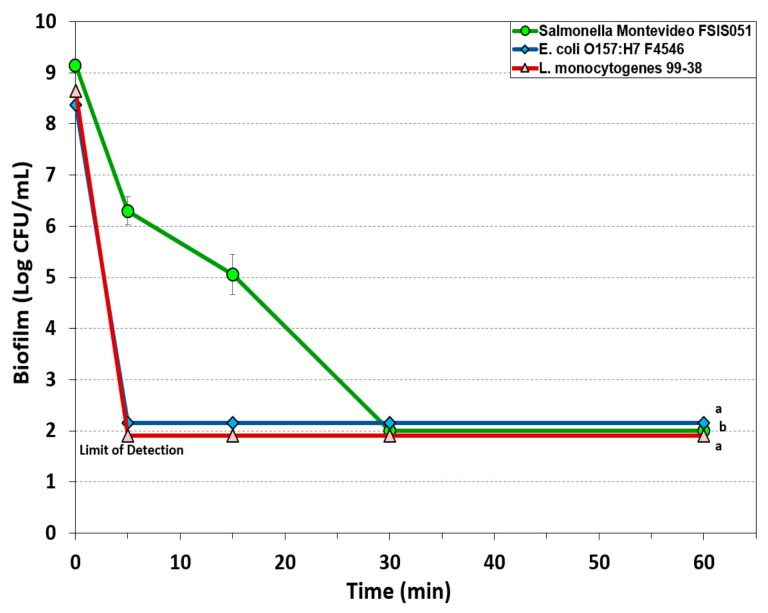
Biofilm microplate lethality assay of extended biofilms of *E. coli* F4546*, S.* Montevideo FSIS 051*, and L. monocytogenes* 99-38 on 7-day biofilms challenged with 500 ppm KC-610 PAA sanitizer for up to 60 min. Data points represent the means of triplicate replications and error bars represent the standard deviations from the mean (some error bars may be hidden by the large symbols). Treatments with different letters are significantly different (RM-ANOVA, *p* < 0.05); treatments with the same letters are not significantly different (RM-ANOVA, *p* > 0.05).

Peroxyacetic acids are becoming more popular for use as sanitizers as microbial problems persist with recurring environmental contamination. We examined KC-610 sanitizer (at 500 ppm) against enhanced biofilms of all three pathogens for durations as long as 60 min (Figure 3). Biofilms of both *L. monocytogenes* 99-38 and *E. coli* O157:H7 F4546 were quickly reduced to below detectable levels (>7.5 log reduction) within 5 min of application, while *S.* Montevideo FSIS 051 was reduced less than 3 logs within 5 min but to undetectable levels by 30 min (Figure 3).

#### 3.1.4. New Generation Quaternary Ammonium Chloride-Based Sanitizers

**Figure 4:**
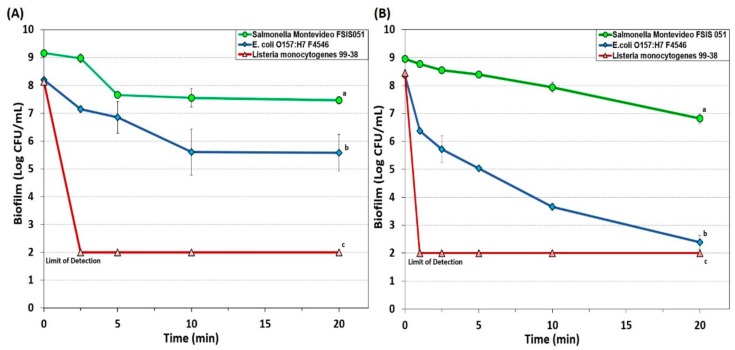
Biofilm microplate lethality assay of extended biofilms of *E. coli* F4546*, S.* Montevideo FSIS 051*, and L. monocytogenes* 99-38 against 7-day biofilms challenged with 5% (**A**) and 10% (**B**) solutions of Sterilex Ultra sanitizer for up to 20 min. Data points represent the means of triplicate replications and error bars represent the standard deviations from the means (some error bars may be hidden by the large symbols). Treatments with different letters are significantly different (RM-ANOVA, *p* < 0.05); treatments with the same letters are not significantly different (RM-ANOVA, *p* > 0.05).

A ‘new generation’ QAC sanitizer, Sterilex Ultra, consists of a 2-part sanitizer including a hydrogen peroxide/QAC solution and an ‘activator’ solution which, after formulation, was used at 5% and 10% strength. Both concentrations acted quickly and rapidly on biofilms of *L. monocytogenes* 99-38, with 5% and 10% formulations reducing *L. monocytogenes* to undetectable levels (>6 log reduction) in 2.5 and 1 min, respectively (Figure 4). Biofilms of *E. coli* O157:H7 F4546 were not affected as much as those of *L. monocytogenes* 99-38, and application of a 5% solution showed a <3 log reduction in 10 min that remained approximately the same through 20 min (Figure 4A), while a 10% formulation slowly decreased *E. coli* O157:H7 F4546 to >6.3 log reduction in 20 min (Figure 4B). *Salmonella* biofilms remained more resistant to Sterilex Ultra, observing only a 1.7 log decrease with 5% formulation (Figure 4A) and a 2.1 log reduction with 10% solution through 20 min (Figure 4B).

**Figure 5:**
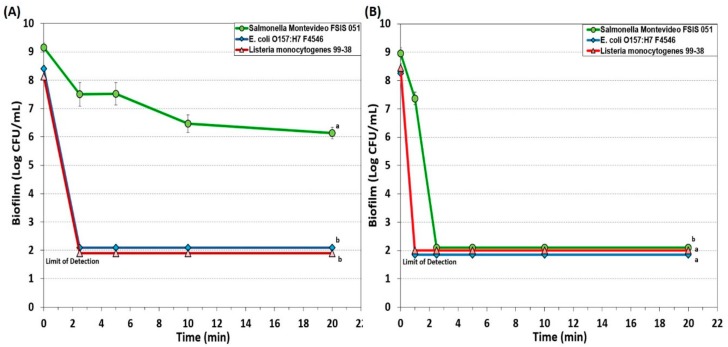
Biofilm microplate lethality assay of *E. coli* F4546*, S.* Montevideo FSIS 051*, and L. monocytogenes* 99-38 7-day biofilms challenged with 5% (**A**) and 10% (**B**) solutions of Decon7 sanitizer for up to 20 min. Data points represent the means of triplicate replications and error bars represent the standard deviations from the means (some error bars may be hidden by the large symbols). Treatments with different letters are significantly different (RM-ANOVA, *p* < 0.05); treatments with the same letters are not significantly different (RM-ANOVA, *p* > 0.05).

Another new generation QAC-based sanitizer was Decon7 that is a 3-part solution formulation consisting of a surfactant (quaternary ammonium compound), an oxidizer (hydrogen peroxide) and an accelerator (diacetin). Decon7 was also used at both 5% and 10% concentrations on biofilms of each of our three pathogens. At 5% solution, Decon7 worked quickly to reduce both *L. monocytogenes* and *E. coli* O157:H7 to undetectable levels (i.e., >6 logs) while *Salmonella* incurred a 3 log reduction and persisted even after 20 min (Figure 5A). When applied at 10% concentration, all three pathogens were reduced to below detectable levels with even the resistant *Salmonella* reaching ~7 log reduction within 2.5 min of treatment (Figure 5B).

### 3.2. Scanning Electron Microscopy of Biofilms of L. monocytogenes 99-38, E. coli O157:H7 F4546, and S. Montevideo FSIS 051

**Figure 6:**
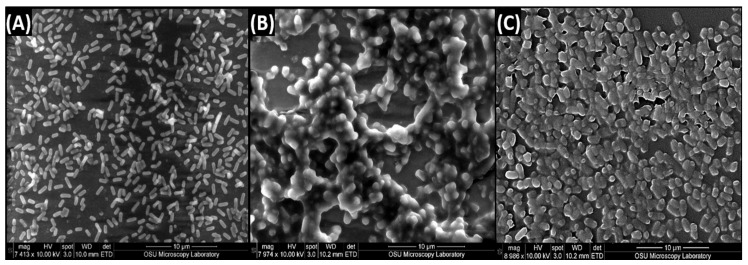
Scanning electron microscopy (SEM) of enhanced 7-day biofilms prepared on slide chambers from (**A**) *Listeria monocytogenes* 99-38, (**B**) *E. coli* O157:H7 F4546, and (**C**) *S.* Montevideo FSIS 051. Approximately 7000–9000-fold magnification.

The 7-day biofilms for *L. monocytogenes* 99-38, *E. coli* F4546, and *S.* Montevideo FSIS 051 were also visibly different when examined by SEM (Figure 6). The *Listeria* looked like clean, smooth bacterial cells (Figure 6A) while the *E. coli* (Figure 6B) and *Salmonella* (Figure 6C) appeared to be coated with a film. Each of these biofilms provided >8 log CFU/mL in 200–300 µL when recovered from microplate wells (with trypsin) and enumerated on petri plates.

## 4. Discussion

During our prior work on developing robust, 7-day enhanced biofilms with three strongly-adherent pathogens, we optimized conditions for biofilm formation (seven consecutive days of washing/renewing growth media), fluorescence substrate (5,6-CFDA selected as the better substrate), and enzymatic recovery (trypsin enzyme) from adhered biofilms [38]. Additional studies had indicated to us that a common sanitizer (Bi-Quat) used in our in-house slaughter facility was not effective on *E. coli* O157:H7 biofilms, nor effective against pre-existing biofilms that had developed on workers’ boots. This provided the impetus to examine the effectiveness of five commercial sanitizers against robust biofilms on our microplate biofilm platform under standardized conditions of use.

Chlorine-based solutions are the most common and inexpensive sanitizers used in food industries and, hence, the efficacy of other sanitizers is often evaluated by comparison with chlorine-based sanitizers [40]. In the USA, chlorine is used for the sanitization of equipment as well as poultry meat, even though reactive chlorine has been shown to generate potentially carcinogenic chlorinated byproducts (trihalomethane, semicarbazide) [41,42]. Because of this, the use of chlorine has been drastically curtailed in other parts of the world, particularly in the European Union, and even in the USA there are strict limits on levels of free chlorine in industrial waste streams.

Commonly used chlorine sanitizing compounds include liquid chlorine, hypochlorites, and chloramines. Chlorines are strong oxidizing agents and broad spectrum germicides which have a variety of modes of action of disinfection. They are found to act on microbial membranes, oxidize sulfhydryl enzymes, hinder DNA synthesis and damage DNA, oxidize respiratory components, inhibit protein synthesis and act by a combination of factors acting simultaneously [43]. However, as oxidizing compounds, they are readily rendered inactive depending on the availability of organic reducing material [44].

The application of 10-Chlor (hypochlorite) at a low concentration was largely ineffective, and the biofilm organic layer may have reduced the low level of oxidizer (hypochlorite) present as 200 ppm (Figure 1A). Increasing the concentration to 1000 ppm overcame the inactivation of the active agent yet still showed differences possibly based on the sensitivity/resistance of the target organisms (Figure 1B).

Quaternary ammonium compounds (QACs) are cationic surface active agents (surfactants) that contain a centrally placed nitrogen atom covalently bonded with four alkyl (R) groups and a negatively charged anion portion [45]. The activity of QACs is the result of cationic charges that form electrostatic bonds with negatively charged bacterial proteins [46] and the application of such antimicrobials involves interaction with membrane proteins, disruption of membrane integrity and leakage of cytoplasmic contents [47]. QACs are stable, active, possess low toxicity and have higher efficacy against Gram-positive bacteria, yeasts, molds, and lipid-containing viruses. They are, however, not as effective against Gram-negative bacteria, endospores, and bacteriophages [45,48]. The nature and length of alkyl (R) groups determine the antimicrobial activity of QACs with a methyl group of 12 to 14 carbon chains showing greater activity [45]. In the USA, CFR Title 21 restricts the use of quaternary ammonia compounds to 200 ppm on food contact surfaces. Bi-Quat is an example of early generation QAC sanitizers that have been widely used in the food industry. In our studies, 200 ppm Bi-Quat was effective against *L. monocytogenes*, but showed limited effectiveness against *E. coli* and *Salmonella* unless used at 1000 ppm (Figure 2).

Peroxyacetic acid (PAA) is also simply known as peracetic acid and is a stronger oxidizing agent than chlorine. Commercially available PAA is the equilibrium form of a quaternary mixture of acetic acid, hydrogen peroxide, PAA, and water [49]. The popularity of PAA as a sanitizer is due to its scope of action against bacteria, yeast, and fungi, its decomposition into harmless byproducts, and its application over a wide range of temperature (0–40 °C) and pH (3–7.5) [48]. Federal regulations prohibit the use of PAA above 200 ppm for food contact surfaces although higher levels may be used if subsequently rinsed with water. The mode of action of PAA, like any other oxidizing agent, is denaturing proteins, dislocating or rupturing the cell wall, and oxidizing sulfhydryls and sulfur bonds in enzymes and other metabolites [50]. Peracetic acid has been found to eliminate viable *S. aureus* (reduction by 98%) and *P. aeruginosa* (99% reduction) on surfaces with only 1 min of contact time but was not effective against the same bacteria in biofilms [51]. In our study, PAA (KC-610) was very effective in reducing *L. monocytogenes* and *E. coli* O157:H7 to undetectable levels (i.e., >6.5 log reduction) and ~3 log reduction of *Salmonella* within 5 min (Figure 3).

Hydrogen peroxide is a clear, colorless liquid and an environmentally friendly (non-toxic) sanitizer widely used in the medical field and in food industries. It is effective against a broad spectrum of microorganisms including viruses, bacteria, bacterial endospores and yeasts [15,50], and the primary mode of action is through oxidization and production of hydroxyl (•OH) free radicals. These free radicals can attack and disrupt membrane lipids, target DNA and proteins (sulfhydryl bonds) and affect other essential cellular components [50]. Hydrogen peroxide is extensively used in produce industries to sanitize the surfaces of whole and fresh cut melons [52]. Activity is further enhanced in combination with new generation QACs, including products like Sterilex and Decon7 that combine the effectiveness of multi-quaternary ammonium compounds with hydrogen peroxide. Our data on extended biofilms showed that Sterilex was very effective against *L. monocytogenes* 99-38 (>6 log reduction in 2.5 min at 5% strength), moderately effective against biofilm of *E. coli* O157:H7 F4546 (<3 log reduction in 2.5 min at 10% strength), and least effective against *S.* Montevideo FSIS 051 (~1 log reduction in 10 min at 10% strength) (Figure 4). Decon7 is similar to Sterilex, but it also includes diacetin (glycerin diacetate) as an ‘accelerator’ which appears to provide additional effectiveness against biofilms, as evidenced by our data, achieving a >6 log reduction in *L. monocytogenes* and *E. coli* O157:H7 within 1 min (10% strength) and >7.5 log reduction in *Salmonella* in 2.5 min (Figure 5).

Examination of the data for the various sanitizers on the biofilms used in this study demonstrates differences between the effect on *L. monocytogenes* 99-38 (most sensitive) vs. *E. coli* F4546 and *S.* Montevideo FSIS 051 (less sensitive) (Figure 1, Figure 2, Figure 3, Figure 4 and Figure 5). When biofilms were examined by SEM, we observed that *L. monocytogenes* 99-38, despite having been enriched by seven consecutive days of washing/growth, were observed as ‘clean’ cells fixed to the surface (Figure 6A). However, those of *E. coli* O157:H7 F4546 and *S.* Montevideo FSIS 051 appeared as if covered with a coating (Figure 6B,C). The results showing less sensitivity to the sanitizers suggests that protection might be afforded by the EPS produced by these organisms (Figure 6). Such structures have been observed by others and not only act as a ‘glue’ that holds the biofilm together, but also as a protective coating that restricts diffusion of nutrients and/or antimicrobials from reaching retained and embedded cells [53,54].

## 5. Conclusions

The array of published data concerning the inactivation of microorganisms with sanitizing disinfectant antimicrobials is lengthy and overwhelming [55,56,57]. Microbial susceptibility to sanitizers can largely depend on whether the cells are loosely available or if they are buried within the intricacies of a biofilm. In order to develop a standardized robust biofilm for testing purposes, we screened for the most strongly adherent stains of three different pathogens, and applied them in an extended microplate biofilm assay by daily removal of planktonic cells and re-application of fresh media daily for 7 days [38]. During our application of commercial sanitizers within this standardized platform, we have observed that some are more effective than others and over short or longer application times (Figure 1, Figure 2, Figure 3, Figure 4 and Figure 5). In all cases, it appears that *L. monocytogenes* 99-38, even when presented as a biofilm, is the most sensitive of the three pathogens we have tested, while *E. coli* F4546 and *S.* Montevideo FSIS 051 are much less sensitive. This could be due to the fact that *L. monocytogenes* does not make EPS (appears as smooth naked cells, Figure 6) while both *E. coli* and *Salmonella* are known to make EPS and are observed as covered with a coating in the SEM images of our biofilms (Figure 6). Comparisons of sanitizers should be applied by a standardized regimen with sufficiently robust biofilms that can readily distinguish differences between biocides.

## Figures and Tables

**Table 1:** Sanitizers used in this study.

Trade Name	Active Ingredients	Use Level	Source
Bi-Quat	Dimethyl ethylbenzyl ammonium chloride (5.1%); Alkyl dimethyl benzyl ammonium chloride (5.1%); Ethanol (1.1%)	200 ppm,	Birko Corp.
		1000 ppm	
10-Chlor	Sodium hypochlorite (<20%); Sodium hydroxide (<5%)	200 ppm,	Birko Corp.
		1000 ppm	
Sterilex solution	1.Ultra Disinfectant Cleaner: Hydrogen peroxide (5.5%–7.2%), Alykl dimethyl ethyl benzyl ammonium chloride (2.5%–3.5%), Alkyl (C12,C14,C16) dimethyl benzyl ammonium chloride (2.5%–3.5%)	5%, 10%	Sterilex Corp.
	2. Ultra Activator Solution: Sodium carbonate (4%–8%); Potassium carbonate (4%–8%); Tetrasodium ethylenediaminetetraacetate (3%–7%)		
KC-610	Peroxyacetic acid (5%–6%), Hydrogen peroxide (25%–58%), Acetic acid (5%–10%)	500 ppm	Packers Chemical
Decon7 solution	1.Quaternary ammonium chloride	5%, 10%	Decon7 Systems
	Benzyl-C12-C16 Alkyl Di-methyl Chlorides (5.5%–6.5%);		
	2. Hydrogen peroxide (<8%);		
	3. Accelerant: Diacetin (30%–60%)		
